# A Rare Case of a Depressed Inner Table Fracture Without an Outer Table Fracture Following Trauma in a 20-Year-Old Male Patient

**DOI:** 10.7759/cureus.105286

**Published:** 2026-03-15

**Authors:** Zaid Khan, Sulaiman Hussain, Ahmad Reshad Payenda, Tariq Khan, Khizar Hayat

**Affiliations:** 1 Neurosurgery, Northwest General Hospital and Research Center, Peshawar, PAK; 2 Medicine and Surgery, Northwest School of Medicine, Peshawar, PAK; 3 Neurological Surgery, Northwest General Hospital and Research Center, Peshawar, PAK

**Keywords:** computed tomography, depressed skull fracture, inner table fracture, parietal bone, skull injuries

## Abstract

Depressed skull fracture (DSF) refers to an inward distortion of the cranium, usually involving both the outer and inner tables, typically arising from blunt head trauma. These fractures can disrupt the meninges and underlying brain parenchyma.

In this case report, we describe an unusual case of a 20-year-old male patient with a depressed skull fracture of the inner table with an entirely intact outer table, with no evidence of epidural, subdural, intraparenchymal hemorrhage, or contusion. The dura mater appeared intact, with no evidence of pneumocephalus or cerebrospinal fluid leakage. Three-dimensional CT reconstructions further confirmed the isolated nature of the inner table depression and the complete preservation of adjacent intracranial structures.

This case report is exceptionally rare, characterized by an isolated depressed inner-table skull fracture with intact outer table, dura, and parenchyma. High-resolution CT with thin reconstructions was essential for diagnosis and guided safe conservative management.

## Introduction

Depressed skull fracture (DSF) refers to an inward distortion of the cranium, typically involving both the outer and inner tables, usually arising from blunt head trauma [1]. DSFs are comminuted cranial vault injuries in which a part of the skull is forced inside the brain, most typically from a focused, high-energy blunt impact [2]. Skull fractures are often characterized by pattern (linear, depression, diastatic, basilar) and wound (closed or open/compound) [3]. According to epidemiologic data, the parietal bone is most frequently affected by DSFs, which typically affect patients between the ages of 16 and 45 [4]. Due to their catastrophic mechanism, these injuries have a higher risk of sequelae (seizure, infection, neurologic deficiency) than simple linear fractures [4]. These fractures can cause disruption of the meninges and underlying brain parenchyma and are typically linked to traumatic factors such as road traffic accidents (RTA).

In contrast, a DSF variant with isolated depression of the inner table and an uninterrupted outer table is highly rare [1]. The pathophysiology of this peculiar injury pattern is probably related to the biomechanical reaction of the skull; a forceful blow might cause tensile strains across the diploe that fail the relatively thin inner table while leaving the outer table intact [1]. Clinically, these fractures can be obscure, as the outside cortex and scalp may appear normal; the interior depression is only visible on high-resolution imaging. Reported cases were detected only after extensive CT evaluation (sometimes with 3D reconstruction) and an MRI of the damaged area [1,5].

Such a case over the Rolandic fissure without outer-table involvement after a gunshot injury was initially reported by Standage (1898) [6]. Almost a century later, Miyake et al. (2016) described a similar injury following a fall, referring to it as tensile stress localized on the inner table [7]. Eom (2021) indicated that inadequate impact energy can damage the thin inner table while sparing the outer layer [1]. Together, these findings indicate the inner table can be preferentially fractured by localized tensile impacts while the surrounding structures remain intact.

Recent literature indicates that many skull fractures can be managed non-surgically if there is no concomitant brain injury. As pediatric patients with isolated skull fractures were discovered, none required advanced neurosurgical intervention; the majority were safely monitored without transfer to a specialized care facility [8]. Similarly, an extensive 10-year pediatric trauma series revealed majority of head injury cases (70.3%) were treated primarily with observation, with outstanding results under non-operative therapy [9].

This case report aims to provide an extensive description of an unusual patient presentation, including two seizure-like episodes and mild scalp edema, along with a depressed skull fracture of the inner table having intact dura and brain parenchyma. The purpose of this case report is to identify this unique presentation, which is a very rare occurrence; only two cases in the literature have been reported as of yet. The purpose of this report is to gather more occurrences of this type to ascertain the cause of this isolated inner table fracture.

## Case presentation

A 20-year-old male patient presented to the emergency department approximately three hours following a road traffic accident (RTA). According to witnesses, the patient experienced a sudden loss of consciousness, followed by a generalized seizure-like episode characterized by acute decerebrate posturing and frothing from the mouth. These resolved within approximately 40-50 minutes, with the patient regaining full consciousness and orientation. No vomiting history was present. Prior to being referred to our hospital, the patient underwent initial advanced trauma life support (ATLS) protocols at a local facility.

Upon arrival at our emergency department, the patient was completely conscious and oriented to time, place, and person, with a Glasgow Coma Scale (GCS) score of 15/15. Neurological assessment revealed no focal abnormalities. Physical examination revealed a localized scalp collection in the parietal region of the skull with an accompanying clavicular fracture. Motor strength, tone, and deep tendon reflexes were all within normal range. The clavicular fracture was treated conservatively by the orthopedics team. Initial laboratory tests were within normal physiological ranges and are summarized in Table 1.

**Table 1:** Initial laboratory tests were within normal physiological ranges.

Parameter	Result	Reference range
Hemoglobin	15.1 g/dL	13.0 - 17.0 g/dL
Total leukocyte count	12 × 10⁹/L	4.0 - 11.0 × 10⁹/L
Platelets	130 × 10⁹/L	150 - 450 × 10⁹/L
Serum sodium	132 mmol/L	135 - 145 mmol/L
Serum potassium	4.68 mmol/L	3.5 - 5.0 mmol/L
Serum chloride	98.7 mmol/L	98 - 107 mmol/L
Urea	44 mg/dL	15 - 45 mg/dL
Creatinine	0.98 mg/dL	0.6 - 1.3 mg/dL
Activated partial thromboplastin time (aPTT)	30.0 s	25 - 35 s
Prothrombin time (PT)	11.0 s	10 - 13 s
International normalized ratio (INR)	1.0	0.8 - 1.2

**Figure 1:**
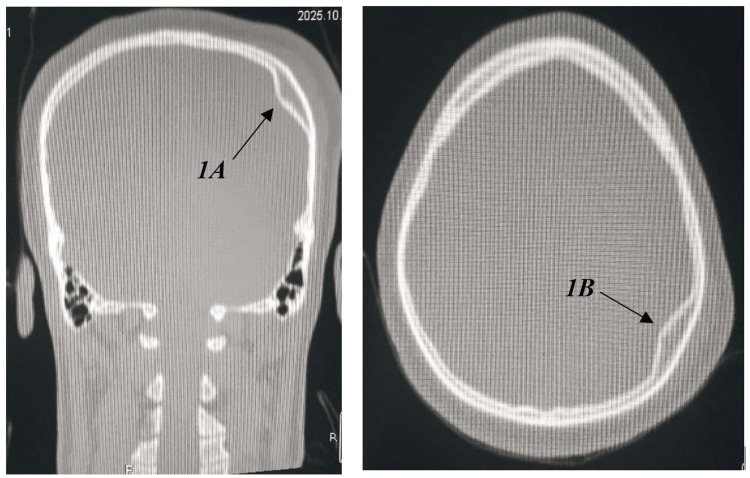
(A) Coronal and (B) Axial non-contrast cranial computed tomography (CT) scans (bone window) revealing a focal depressed fracture limited to the inner table of the left parietal bone (arrows). Note the complete preservation and intactness of the outer table and diploic space.

**Figure 2:**
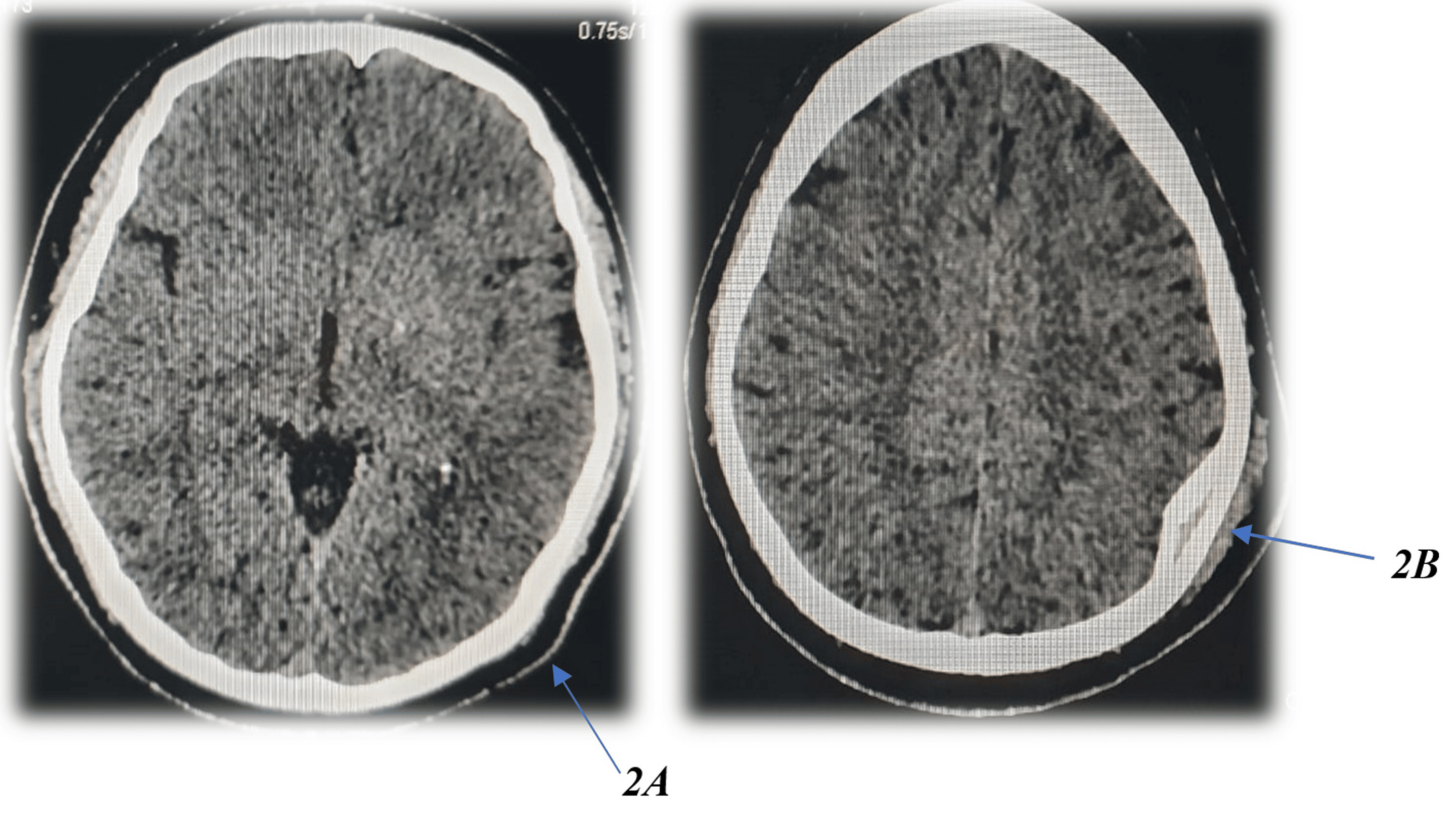
(A,B) Axial non-contrast cranial computed tomography (CT) scans (tissue window) demonstrating intact brain parenchyma, normal ventricular size, and absence of epidural, subdural, or intraparenchymal hemorrhage or contusion.

**Figure 3:**
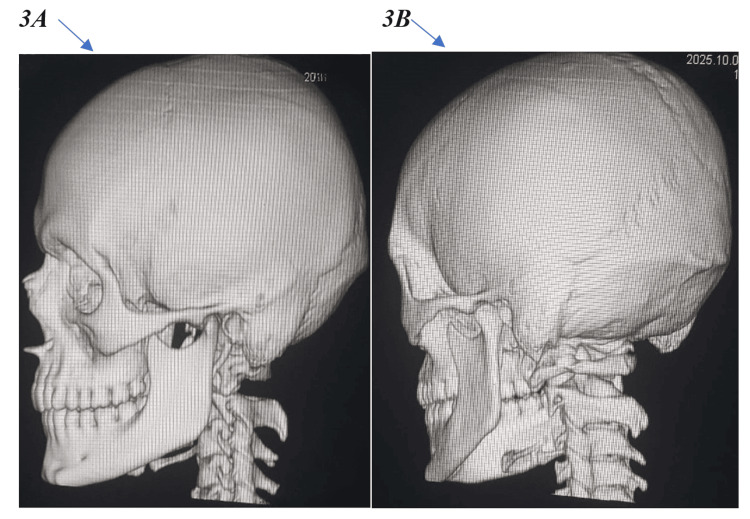
(A, B) Three-dimensional computed tomography (CT) reconstructions of the skull (lateral views) providing a comprehensive visualization of the calvarium. These images confirm the absence of any visible fracture or depression of the outer table, highlighting the isolated nature of the inner table injury.

**Figure 4:**
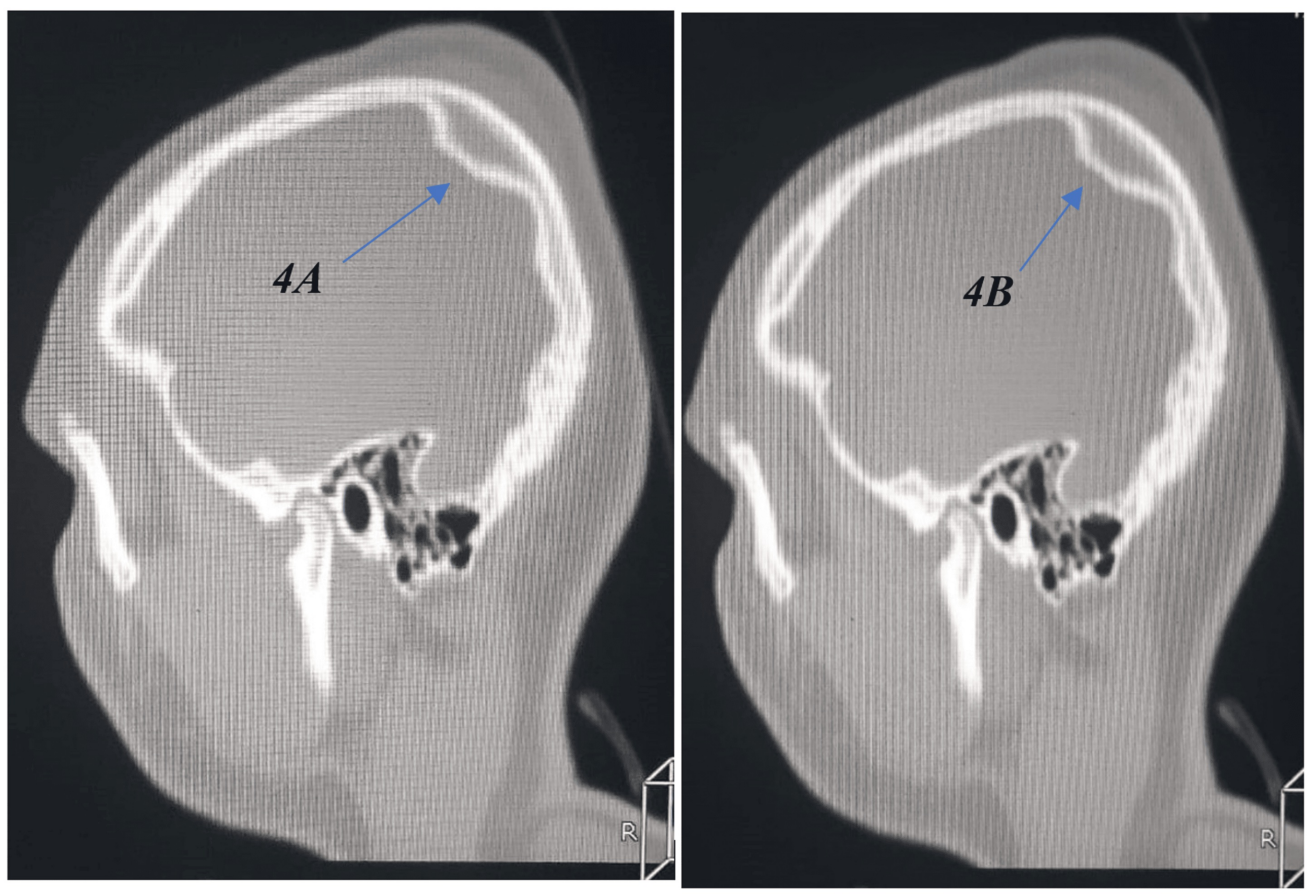
(A, B): Sagittal CT scan (bone window) further delineating the intact outer table and the subtle depression of the inner table.

Non-contrast cranial computed tomography (CT) was performed using a Toshiba multidetector scanner (Canon Medical Systems Corporation, Otawara, Japan) with standard slice thickness 5 mm, reconstruction 1 mm and processed with ALEX VON software under both standard and bone-window settings, unequivocally indicating a depressed fracture precisely in the inner table of the left parietal bone, measuring approximately 32.1 mm in length, and 9.5 mm in depth (Figure 1). Interestingly, the outer table is entirely intact, with no evidence of epidural, subdural, intraparenchymal hemorrhage, or contusion (Figure 2). The dura mater appeared intact, with no evidence of pneumocephalus or cerebrospinal fluid leakage. Three-dimensional CT reconstructions further confirmed the isolated nature of the inner table depression and the complete preservation of adjacent intracranial structures (Figure 3). Sagittal CT images in the bone window (Figure 4A-4B) further delineated the intact outer table and the subtle depression of the inner table, providing additional confirmation of the lesion’s confined nature.

Management plan

Upon admission, the patient was promptly placed under intensive neurological observation based on a history of momentary loss of consciousness, seizure-like activity, and decerebrate posture. The initial management strategy focused on supportive care and subsequent brain damage prevention. Intravenous fluids were initiated with 0.9% sodium chloride to preserve euvolemia and cerebral perfusion. Prophylactic anticonvulsant therapy was initiated with IV levetiracetam (500 mg BID) to mitigate the risk of post-traumatic seizures, especially given the history of initial seizure-like episodes. Pain was managed with paracetamol (PROVAS IV) 500mg TDS. Intravenous esomeprazole (RISEK) was administered to prevent stress ulcers.

Meanwhile, shoulder immobilization was employed as a conservative treatment for the concomitant clavicular fracture. Regular neurological examinations were carried out, and the patient was continuously monitored for any signs of intracranial pressure changes or neurological deterioration. Given the absence of dural injury, brain parenchymal injury, or significant mass effect on the CT scan, non-operative treatment of the depressed inner-table fracture was commenced. The patient's hemodynamic condition remained stable throughout the hospitalization, with no recurrence of seizure activity or neurological impairments.

## Discussion

DSFs are common manifestations of blunt head trauma, typically spanning both the outer and inner tables of the calvarium, often accompanied by dural perforation and underlying brain parenchymal damage [10]. The case herein reveals an exceptionally rare variation, a depressed fracture of the inner table with a fully intact outer table, dura mater, and brain parenchyma.

In 1898, Standage documented the first known case of an isolated inner table fracture [6]. However, an epidural clot caused a major inner table fracture and consequent neurological impairments. Furthermore, Standage observed no dural wound or bleeding beneath the dura, consistent with our patient's intact dural integrity [6]. Even so, the cause of injury, a gunshot, and deteriorating neurological outcome in Standage's case [7], was substantially different from the blunt trauma and swift neurological recovery reported in our patient.

More recently, Miyake et al. (2016) identified an isolated inner table DSF soon after a fall, attributing the fracture to the inner table's severe tensile stress [7]. This biomechanical interpretation is validated by computer simulations, implying that although compressive forces are applied to the outer table at impact, the inner table can be exposed to immense tensile forces, yielding a preferential fracture [7]. Eom (2021) conferred credence to this concept, illustrating an identical case following a motorcycle accident, suggesting that the insufficient impact energy could harm the thin inner table while preserving the stronger outer table [1]. However, Eom's case comprised a damaged diploe and a hemorrhagic cranial contusion, distinguishing it from our patient, who presented no indications of parenchymal and meningeal injury on the CT scan of the skull.

Our patient's presentation is characterized by an isolated inner table fracture with the entire preservation of the outer table, dura mater, and brain parenchyma, which corresponds to a more atypical pattern. The momentary neurological signs, featuring decerebrate posture and seizure-like activity, are particularly striking considering the patient's rapid and complete recovery. This indicates an initial blow, resulting in a focal inner table depression. The energy transfer was localized precisely, preventing direct damage to the underlying structures or severe mass effects. The absence of dural rupture and brain parenchyma injuries vastly lowers the severity of complications such as cerebrospinal fluid leak, infection, and post-traumatic epilepsy, which frequently occur in classic DSFs [11,12].

Our case is very relevant to the biomechanical description of the tensile stress prevailing on the inner table. The skull dynamically responds to blunt force trauma, as the inner table is thin and more brittle and prone to tensile stress [10]. In high-velocity impacts, such as the road traffic accident that our patient experienced, the skull may distort, resulting in tensile stress on the inner table and compression on the outer table. If the impact force is inadequate to exceed the outer table compressive strength while exceeding the inner table tensile strength, an isolated inner table fracture can occur. The transient neurological symptoms seen in our patient could be caused by the temporary distortion of the skull and momentary cerebral concussion without structural brain damage, in contrast to direct parenchymal damage from the depressed fragment [10].

This case illustrates the relevance of high-resolution imaging, especially thin-slice CT with bone windows, in accurately diagnosing isolated inner-table skull fractures. Thin (1-1.5 mm) CT bone-window reconstructions are recommended to optimally visualize subtle skull fractures [13].

A comprehensive assessment of dural and parenchymal integrity is essential for making management decisions. In cases like this, where cerebral structures are preserved, the conservative approach is opposed to DSFs with dural invasion or a large mass effect secondary to a contusion or hematoma, which often require more drastic operative surgery [14,15].

## Conclusions

In conclusion, this case report depicts an extremely unusual incident of an isolated inner table depressed skull fracture with intact dura mater, brain parenchyma, and outer table. It contributes to the limited literature on this unique injury, reaffirming the biomechanical theory of tensile stress as a contributory factor and illustrating the significance of precise diagnostic imaging. Despite exhibiting significant symptoms at first, our patient's positive neurological outcomes suggest that such injuries, when unaccompanied by intracranial injuries, may progress in a benign manner, though careful monitoring remains paramount.
